# Obstetrical fractures of the femur in developing countries: Predisposing factors and therapeutic approach

**DOI:** 10.4103/0971-9261.71743

**Published:** 2010

**Authors:** Gabriel Ngom, Mbaye Fall, Issa Amadou, Désiré Alumeti Munyali

**Affiliations:** Department of Pediatric Surgery, Hospital Aristide Le Dantec, Dakar, Senegal

Sir,

Obstetrical fracture of the femur is uncommon.[1] It ranks third among obstetrical fractures of bones, after the fractures of the clavicle and the humerus.[2] The main risk factors identified are breech delivery, caesarean section, macrosomia, primiparity, twin pregnancy and fetal osteoporosis.[3,4]

Treatment for this condition uses three methods: spica cast, Pavlik harness and Bryant traction.[1,5] Each method has advantages and drawbacks, but the results are overall good and equivalent.

In Senegal, this fracture ranks second in position after the fracture of the clavicle. Between January 1996 and December 2007, 51 neonates presented with 52 obstetrical fractures of the femur, which account for 7.5% of all femoral fractures noted in children. Ours’ is the largest ever reported series in the literature. Risk factors identified were breech delivery, macrosomia, twin pregnancy and osteogenesis imperfecta.

Primiparity was not identified as a risk factor because most women were multiparous (70%). However, we have identified an additional risk factor represented by home delivery. This practice has caused 33.3% of obstetrical fractures. It is performed at home by women who received no health training. This situation is common because most of the population is poor and cannot afford safe delivery.

Spica cast is not readily performed in our department because it requires general anesthesia, which can be hazardous to a newborn in a developing country where pediatric anesthesia is not well controlled. Pavlik harness is not readily available in our daily practice. We use Bryant traction for 10 days supplemented by spica cast for 2 weeks. Our results are excellent: no skin complications or vascular and nervous complications or malunion.

In conclusion, obstetrical fractures of the femur are not an exceptional occurrence in our context. One-third of these fractures are caused by home delivery performed by unskilled women. Treatment involves Bryant traction followed by spica cast, which seems to be the best method in our developing countries, especially in terms of safety for neonates.
